# Indications for a vertical transmission pathway of piscine myocarditis virus in Atlantic salmon (*Salmo salar *L.)

**DOI:** 10.1111/jfd.12990

**Published:** 2019-03-28

**Authors:** Britt Bang Jensen, Stian Nylund, Julie Christine Svendsen, Paul‐Martin R. Ski, Harald Takle

**Affiliations:** ^1^ Section for epidemiology Norwegian Veterinary Institute Oslo Norway; ^2^ Pharmaq Analytiq AS Bergen Norway; ^3^ SalMar Farming Frøya Norway; ^4^ Marine Harvest ASA Bergen Norway; ^5^ Present address: Cermaq Group AS Oslo Norway

**Keywords:** cardiomyopathy syndrome, epidemiology, piscine myocarditis virus, vertical transmission

## Abstract

Losses due to cardiomyopathy syndrome (CMS) keep increasing in salmon‐producing countries in the North‐Atlantic. Recently, Piscine myocarditis virus (PMCV) has been detected in post‐smolts shortly after sea‐transfer, indicating a possible carry‐over from the hatcheries. In addition, there are reports of prevalences of PMCV as high as 70%–90% in certain groups of broodfish, and a recent outbreak of CMS in the Faroe Islands has been linked to the importation of eggs from a CMS‐endemic area. Thus, there is a need to investigate whether PMCV can be transmitted vertically from infected broodstock to their progeny. In the present study, samples from eggs, larvae, fingerlings and presmolt originating from PMCV‐positive broodstock from two commercial Atlantic salmon producers were tested for PMCV. The prevalence of PMCV in the broodstock was 98% in the hearts, 69% in the roe and 59% in the milt. Piscine myocarditis virus was detected in all stages of the progeny until and including the 40 g stage. Piscine myocarditis virus was also detected in presmolt sampled for tissue tropism. This provides farmers with several options for minimizing the risk of transfer of PMCV from broodstock to progeny, including screening of broodstock and aiming to use only those that are negative for PMCV or have low levels of virus.

## 1 INTRODUCTION

Piscine myocarditis virus (PMCV) was identified as the causative agent for cardiomyopathy syndrome (CMS) in 2010 (Haugland et al., 2011; Løvoll et al., 2010). Cardiomyopathy syndrome is an economically important disease that affects Atlantic salmon (*Salmo salar L.*; AS) in marine aquaculture. It was first reported in Norway in 1988 and has been reported from Norway, Scotland, Ireland and the Faroe Islands (Garseth, Fritsvold, Svendsen, Bang Jensen & Mikalsen, 2018).

The disease mainly affects the spongious myocardium, with lesions usually appearing first in the atrium and progressing to the ventricle, and can lead to heart failure and even rupture of the atrial wall (Garseth et al., 2018). Clinical outbreaks of CMS with associated mortalities have primarily been reported from fish in their second year at sea, and the median time reported from sea‐transfer to initial diagnosis of CMS in Norwegian aquaculture is 16 months (Bang Jensen, Brun, Fineid, Larssen & Kristoffersen, 2013). Outbreaks of CMS have, however, been reported in post‐smolt at 2–300 g size, and recent studies have shown that PMCV can be found within the first month after sea‐transfer, at levels below commonly used cut‐off diagnostic values (Hjeltnes et al., 2016; Svendsen, Nylund, Kristoffersen, Takle, Fossberg Buhaug & Bang Jensen, 2019)

To date, clinical outbreaks of CMS have not been reported in fry, smolt or broodfish in the freshwater phase. Piscine myocarditis virus has, however, been detected in broodfish after transfer from sea to freshwater. In a study by Wiik‐Nielsen, Ski, Aunsmo, & Løvoll (2012), PMCV was detected in 16 out of 20 tested broodfish before stripping, and Pharmaq Analytiq reports that certain broodfish groups submitted for analysis can have a prevalence as high as 70%–90% (S. Nylund, *personal observation*). Low levels of viral RNA from PMCV have also been detected in fry (Wiik‐Nielsen et al., 2012).

There is consensus that the primary route of transmission of PMCV is horizontal, and several studies have shown that PMCV and CMS are transmitted between fish in tanks and between farms in the field (Bang Jensen et al., 2013; Fritsvold et al., 2009; Haugland et al., 2011). However, a recent outbreak of CMS in the Faroe Islands has been linked to eggs imported from Norway (Garseth et al., 2018), and in light of the high prevalence of PMCV among broodfish, and the findings of PMCV early after sea‐transfer, a discussion on whether PMCV can be transmitted from broodstock to progeny has been rekindled. Previously, one small‐scale study exploring this possibility was performed, in which PMCV was detected at low levels from fertilized eggs and fry, but not in hatchlings after first feeding (Wiik‐Nielsen et al., 2012). Thus, the results were not sufficient to form a conclusion on whether vertical transmission of PMCV can or does occur. The consequences of a vertical transmission pathway of PMCV for trade and control could be large, and thus, it is important to ascertain whether this is possible or not.

The aim of the present study was to ascertain whether PMCV can be transmitted from broodstock to their progeny, under normal production systems by sampling from commercial producers in the field.

## 2 MATERIALS AND METHODS

### 2.1 Fish cohorts

Fish cohorts from two commercial AS producers in Norway were included in this study. Cohort X includes broodfish and eggs and progeny from these fish. The broodstock for this cohort originated from a seafarm which had experienced some mortality due to CMS in the last half year before stripping. Similarly, cohort Y includes broodfish and eggs and progeny from these fish.

**Figure 1:**
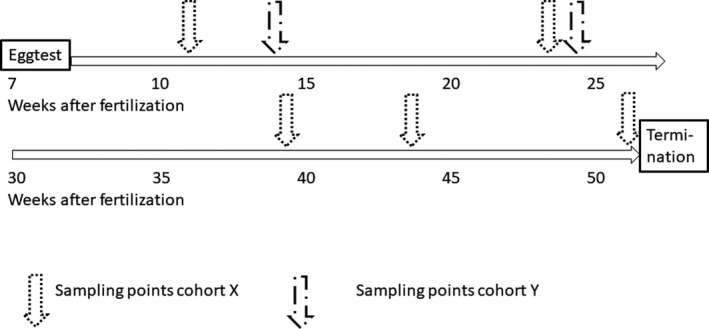
Sample points for the samples taken from cohort X and Y in the study

Samples were taken at different timepoints as depicted in Figure 1 and described below.

### 2.2 Broodstock sampling

Broodstock from both cohorts were stripped in November 2015. At this time, heart (ventricle) and ovarian fluid were sampled from female broodstock. At the same time, milt was sampled from the males used for fertilization from cohort X, and both milt and heart from males from cohort Y. A minimum of 0.2 ml and maximum 1 ml of ovarian fluid or milt were sampled from each fish and stored individually in test tubes without any added media. These samples were refrigerated and kept cold during overnight delivery to the laboratory. All samples from broodfish were analysed by RT‐PCR (see description below), and based on the results, broodfish and their corresponding progeny were sorted into groups A to G as presented in Table 1. In group A and D, both parents were positive for PMCV in both hearts and sexual products. In group B and F, the female was positive for PMCV in heart and ovarian fluid and the male negative for PMCV in milt (Group B) and only weak positive in heart (Group F). In group G, the female was negative in both samples and the male positive in both. In cohort X, there were no females that where negative for PMCV in the heart; therefore, the equivalent to group G consisted of a female which was negative for PMCV in the ovarian fluid and a male with PMCV‐positive milt (group C). Finally, group E was similar to group C, except that the male was also positive for PMCV in the heart.

**Table 1:** Overview of the egg batches in each of cohort X and Y

Group	PMCV in female broodfish		PMCV in male broodfish		Egg batches
	Heart	Ovarian fluid	Heart	Milt	
Cohort X					
A	PMCV +	PMCV ++	Nd	PMCV +	A1, A2
B	PMCV +	PMCV +	Nd	PMCV −	B1, B2, B3, B4
C	PMCV +	PMCV −	Nd	PMCV +	C1, C2, C3, C4
Cohort Y					
D	PMCV +	PMCV +	PMCV +	PMCV (+)	D1, D2, D3, D4
E	PMCV +	PMCV −	PMCV +	PMCV +	E1, E2, E3,
F	PMCV +	PMCV +	PMCV (+)	PMCV−	F1, F2, F3
G	PMCV −	PMCV −	PMCV +	PMCV +	G1^a^, G2, G3

a For this batch, the heart of the female was PMCV +, and the milt from the male was PMCV −

### 2.3 Egg sampling and disinfection

After fertilization, each batch of eggs (a batch meaning the progeny of one male × one female) was kept separate. Eggs from cohort X were sampled before and after disinfection. These eggs were sent in batches together with the broodfish samples to the laboratory. At the laboratory, 50 eggs from each batch were frozen individually before testing. Eggs from both cohorts were disinfected with Buffodine according to manufacturer's protocol: 100 ml Buffodine per 10 L of water for 10 min, followed by five times of rinsing in a dilution of 90 g salt in 10 L freshwater. The eggs were then incubated in single incubators containing one batch of eggs each. From each of the groups A to G as described above, two to four egg batches were selected for further sampling. An overview of the batches included is presented in Table 1.

From two egg batches from group A and four egg batches from each of group B and C, approximately 30 of the eggs that were sampled before disinfection and 30 of the eggs sampled after disinfection were tested with RT‐PCR for PMCV. Each egg was processed and tested individually.

### 2.4 Sampling of progeny

The following sampling and testing were performed from cohort X: From group A, B and C, the egg batches with the highest prevalence of PMCV after disinfection were included in the subsequent study. After approximately 330degree days (in January 2016), the eggs were shocked. At the same time, approximately 120 eggs from each of the three egg batches were sampled and analysed individually. The eggs hatched in February 2016, and samples were taken from each of the three groups four times from hatching until termination right before sea‐transfer, as illustrated in Figure 1. At the last sample point, additionally nine fish from group B were sampled for a study on tissue tropism.

The following sampling and testing were performed from cohort Y: In January, when the eggs had become eyed eggs, 30 eggs were sampled and tested from each of four batches of group D and three batches of group E, and 60 eggs from three bathes from each of groups F and G (see Table 1).

In February, after approximately 110 degree days, 60 yolksac fry were sampled from the egg batch with highest prevalence of PMCV from the previous test from each of group D, E, F and G. All sampled fry were tested individually for PMCV by RT‐PCR. After first feeding, the hatchlings were transferred to another hatchery, where all fry from each of the groups D to G were merged in each of four tanks.

A final sampling was performed in May, when the fry were approximately 6 g. At this time, 90 fry were sampled from each of the four groups. An overview of all sampling points is presented in Figure 1.

#### 2.4.1 Sampling

All samples were kept on RNA‐later until analysis, except milt and roe which were not kept on RNA‐later. From broodstock, pieces of hearts (ventricles) were sampled together with roe and milt during stripping. Eggs were placed in individual containers and tested separately. Yolksac fry were placed whole in containers, whereas the head region (including gills, heart and kidney) was sampled from larger hatchlings/fry. At the last sampling point for cohort X, six tissues were sampled from each of nine fish, in addition to the regular sampling. From these fish, heart, liver, spleen, kidney, brain and gill were sampled separately, in order to study tissue tropism.

#### 2.4.2 Real‐time RT‐PCR

Tissue samples were processed using a Qiagen's Universal Biorobot, with the compatible RNA purification kit (RNeasy 96 Universal Tissue Kit), according to the manufacturers’ recommendation. Extracted total RNA was eluted in a final volume of 100 µl of the supplied kit elution buffer.

Extracted RNA from tissues of salmon was tested by Taqman real‐time RT‐PCR (qScript XLT 1‐ Step RT‐qPCR ToughMix, Quantabio). During the real‐time RT‐PCR screening a house‐keeping gene, elongation factor 1 alpha (EF1A) was used as an internal control (Olsvik, Lie, Jordal, Nilsen, & Hordvik, 2005), and a specific assay was used for detection of PMCV (Nylund et al., 2018). The primer and probe concentrations had been optimized and found to be 900 nM for all primers used, and 225 nM for the corresponding probes. The samples were run in simplex for the internal control, and triplicates for detection of PMCV in standard 384‐well plates. All assays were run in a total volume of 10 µl in each well, with 2.5 µl of isolated total RNA as the template. Plates were analysed in an Applied Biosystems 7900 HT real‐time machine under standard conditions. Each run consisted of 45 cycles, and the samples were considered positive when the fluorescence signal increased above a set threshold of 0.09. The PMCV assay has a repeatable cycle threshold value (Ct) of 34.7 (Svendsen et al., 2019), and average Ct‐values were calculated based on the results from all values found in the triplicates for each sample. Criteria for determination of this average Ct‐value are normally based on the requirement that a minimum of two out of three in a triplicate are below the repeatable cycle threshold value. For the purpose of this study, an average of all Ct‐values was included in the calculation, regardless of Ct‐value.

### 2.5 Statistical analysis

For each batch of samples, the prevalence of PMCV with confidence intervals was calculated using the binom.test in R (R Core Team, 2018).

## 3 RESULTS

### 3.1 Broodfish

The results of the PCR‐tests of broodstock are shown in Table 2. The prevalence of PMCV in heart tissue was 98.4%, with only two samples testing negative. In roe and milt, the prevalence was 69.3% and 58.8%, respectively. The viral levels in heart tissue were generally medium to low (median Ct‐values of 25.7–31.5), whereas they were low in roe and milt (Ct‐value medians of 36.3–36.9).

**Table 2:** Results from PCR‐tests of samples from broodstock of Atlantic salmon (*Salmo salar*) from cohort X and Y for piscine myocarditis virus (PMCV)

	No. of samples	No. of positive samples	Ct‐value range (median)
Cohort X			
Females			
Heart	65	65	14.2–34.4 (25.7)
Roe	67	43	34.6–40.55 (36.9)
Males			
Milt	46	28	35.4–37.6 (36.6)
Cohort Y			
Females			
Heart	60	58	15.4–36.5 (27.0)
Roe	60	45	33.0–39.1 (36.3)
Males			
Heart	5	5	25.6–34.8 (31.05)
Milt	5	2	36.7–36.9 (na)

### 3.2 Disinfection

**Figure 2:**
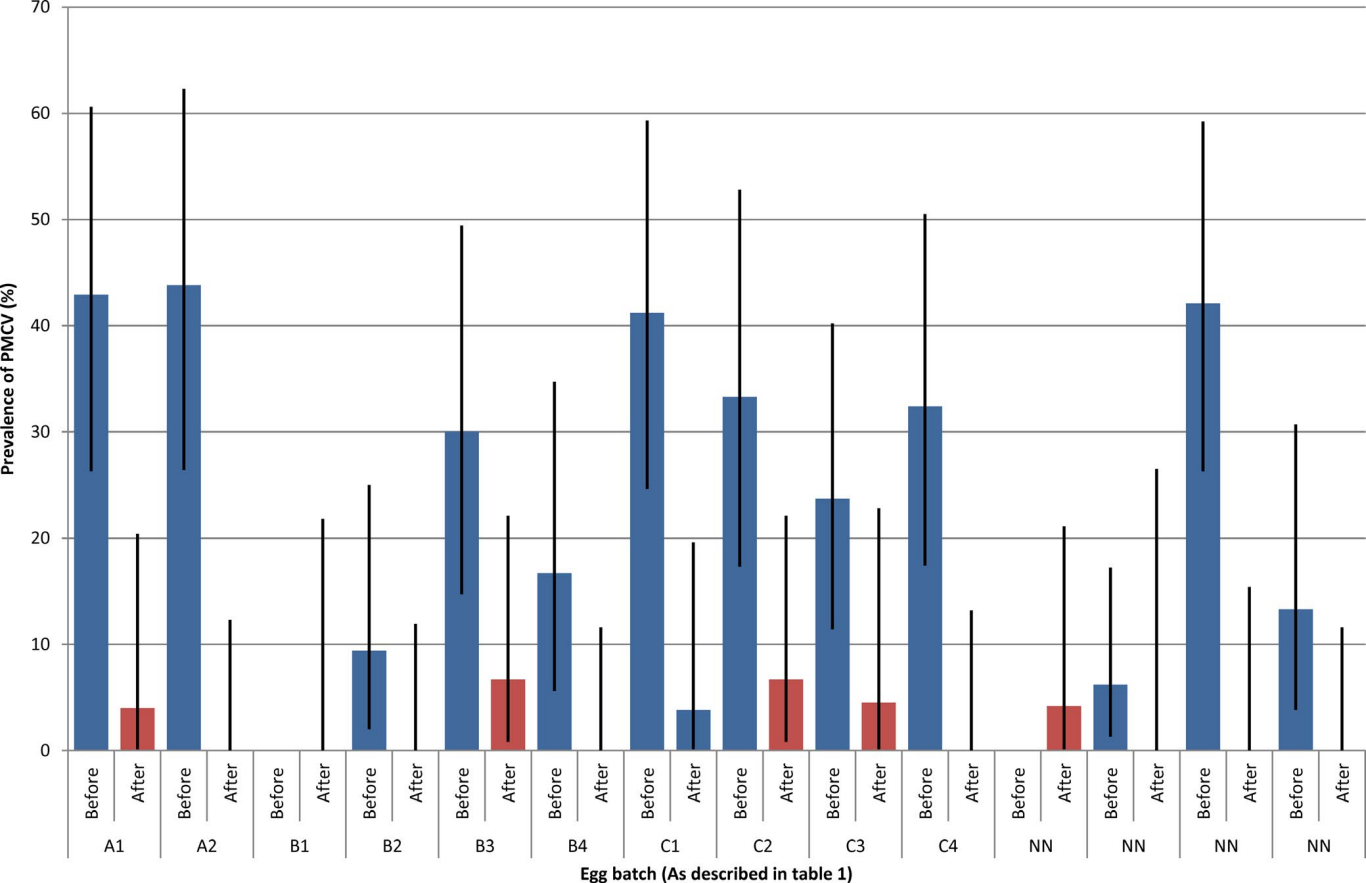
Prevalence of Piscine myocarditis virus (PMCV) in egg batches tested before (blue) and after (red) disinfection. Black lines indicate 95% confidence intervals based on sample size

PMCV was detected in all egg batches from cohort X before disinfection, with an overall prevalence of 27.5%. Prevalence in individual batches ranged from 6.2% to 43.8% (Figure 2). Ct‐values from these samples were between 36.0 and 42.0. After disinfection, PMCV was detected in six of the 14 batches tested, with prevalences from 4%–6.7%. Ct‐values ranged from 37.0 to 41.3 in these samples.

### 3.3 Progeny

**Figure 3:**
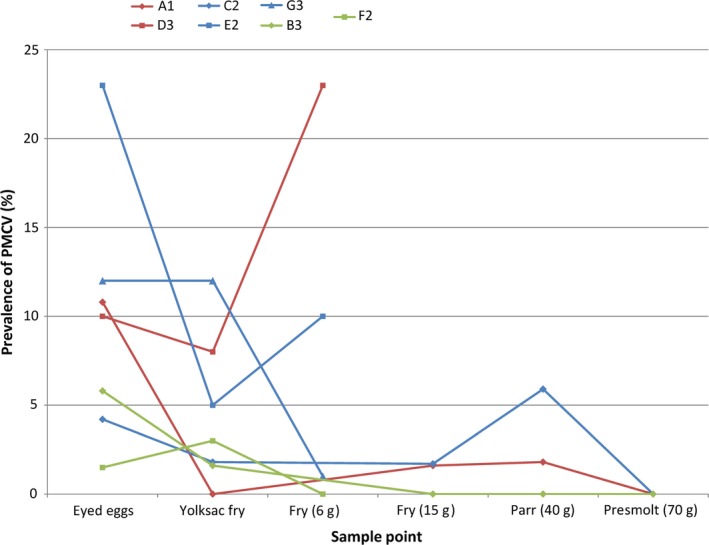
Prevalence of Piscine myocarditis virus (PMCV) in seven egg batches from cohort X and Y, sampled at different timepoints (see also Tables 3 and 4). Red lines indicate batches where both milt and roe from the parents were PMCV positive, blue lines indicate batches where milt was positive and roe negative, and green lines indicate batches where milt was negative and roe positive (see also Table 1)

The results from testing of cohort X for PMCV at different time points are presented in Table 3 and Figure 3. At the first sample point, the prevalence in each of the egg batches tested varied from 4.2% to 10.8%, with confidence intervals from 1.4% to 17.8%. At the second and third sample point (larvae and fry stage), the prevalence was between zero and 1.8%, with confidence intervals from 0% to 9.6%. At the 40 g stage, the prevalence varied from zero to 5.9% (CI 0%–14.4%). At 70 g, we were not able to detect any PMCV in any of the groups from cohort X. The Ct‐values of all positive samples were between 35.4 and 37.8, except from one sample from 15 g fry which had a Ct‐value of 33.9. In 6 of the 9 fish that were sampled for tissue tropism at 70 g, PMCV was detected in either heart, liver or spleen with Ct‐values between 34.6 and 36.9. The other five organs tested in each fish were negative, as was the remaining three fish.

**Table 3:** Results from testing of egg batches from cohort X at different timepoints. CI = 95% Confidence interval based on sample size

Cohort X	Shocked eggs			Larvae			Fry (15 g)			Parr(40 g)			Presmolt (70 g)		
Egg batch	No of samples	Prevalence (%)	CI	No of samples	Prevalence (%)	CI	No of samples	Prevalence (%)	CI	No of samples	Prevalence (%)	CI	No of samples	Prevalence (%)	CI
A1	120	10.8	5.9–17.8	68	0	0–5.3	63	1.6	0–8.5	55	1.8	0–9.7	60	0	0–6.0
B3	120	5.8	2.4–11.6	62	1.6	0–8.7	67	0	0–5.4	54	0	0–6.6	60	0	0–6.0
C2	120	4.2	1.4–9.5	56	1.8	0–9.6	60	1.7	0–8.9	68	5.9	1.6–14.4	60	0	0–6.0

The prevalence of PMCV in the eyed eggs from the 13 egg batches tested from cohort Y varied from 1.7% to 23.3%, with confidence intervals from 0 to 42.3 (Table 4). In the four batches tested at the yolksac fry stage, the prevalence was between 3.3% and 11.7% (CI from 0.4 to 22.6). At the last sampling point at 6 g, the prevalence varied from 0% to 23.3%, with confidence intervals from 0 to 33.4. From Figure 3, it can be seen that the prevalence was low in group F2 at all sample times, and this group was the only one found negative at the last sample time. The Ct‐values of the positive tests were within the range of 35.8–39.3, except for one sample with Ct of 31.4 in group E at the last sample point, and 4 samples with Ct‐values from 27.8 to 34.1 in group F at the same sample point.

**Table 4:** Results from testing of egg batches from cohort Y at different time points. CI = 95% confidence interval based on sample size

Cohort Y	Eyed eggs			Yolksac fry			Fry (6 g)		
Egg batch	No of samples	Prevalence (%)	CI	No of samples	Prevalence (%)	CI	No of samples	Prevalence (%)	CI
D1	30	3.0	0.1–17.2		–				
D2	30	6.7	0.8–22.1		–		90	23.3	15.1–33.4
D3	30	10.0	2.1–26.5	60	5.0	1.0–13.9			
D4	30	20.0	7.7–38.6		–				
E1	30	20.0	7.7–38.6		–				
E2	30	23.3	9.9–42.3	60	8.3	2.8–18.4	90	10.0	4.7–18.1
E3	30	10.0	2.1–26.5		–				
F1	60	10.0	3.8–20.5		–				
F2	60	1.7	0–8.9	60	3.3	0.4–11.5	90	0	0–4.0
F3	60	10.0	3.8–20.5		–				
G1	60	10.0	3.8–20.5		–				
G2	60	10.0	3.8–20.5		–		90	1.1	0–6.0
G3	60	11.7	4.8–22.6	60	11.7	4.8–22.6			

In Figure 3, results from the seven batches from both cohort X and Y that were sampled over time are shown. The two batches with persistently low prevalence of PMCV both originate from eggs where the milt was negative and the roe positive for PMCV. In all other batches, the milt was positive for PMCV, and from the figure it does not seem as if there was a difference between those batches were the roe was also positive for PMCV and those in which it was not.

## 4 DISCUSSION

In this study, we have found PMCV in both hearts and sexual products of broodfish, and in their progeny at several stages of development. This indicates a possible pathway for transmission from broodstock to smolt.

The prevalence of PMCV in the broodfish was very high, with positive real‐time RT‐PCR results from 128 hearts of the 130 tested. There were no clinical signs of CMS at the time of sampling, and the Ct‐values in the hearts were also higher than what is normally seen at clinical outbreaks of CMS. A recent study has shown that fish groups that have gone through a clinical outbreak of CMS remain positive for PCR‐testing until the time of slaughter (Svendsen et al., 2019). The broodfish that were used in the present study had gone through a clinical outbreak earlier, so the findings most likely indicate a carrier‐state. The prevalence of PMCV in the tested roe (69.3%) and milt (58.8%) was high, but the Ct‐values in the positive samples were all just below or just above the diagnostic cut‐off value of 34.7. This diagnostic cut‐off value is calculated by determining the lowest amount of template in a sample that can be consistently detected and reproduced, using the same reaction conditions (Sloan, 2007). For the purpose of using real‐time RT‐PCR as a diagnostic tool for detection of viruses, the cut‐off value that is normally employed is important to ensure that any positive detections of a virus is consistent when comparing both intra laboratory runs, and analyses performed on the same sample in different laboratories. This is also a hallmark of research, where scientists should be able to reproduce results performed by others to confirm the findings. The disadvantage of this strict approach is that relevant information regarding prevalence and presence of a virus in carrier status individuals can be lost. To compensate for this, studies that include values above the cut‐off values should always include relevant controls to ensure that positive detection occurs as a result of amplification of the intended target and also include enough samples in the study to ensure that the overall trends in the results are shown to be consistent. In this study, to ensure that any positive samples appeared as a consequence of detection of PMCV gene sequences, and not as an artefact of the real‐time RT‐PCR reaction parameters, all runs were performed with negative RNA extraction controls and with the use of positive controls. The negative controls were subjected to the same extraction protocol and reaction parameters as the tissue samples, and were consistently negative in all analyses. This indicated that positive signals above the repeatable threshold for PMCV in tissue occurred as a result of amplification of viral sequence, and not as consequence of either unspecific amplification or degeneration of the fluorescent dye from the PMCV probe.

In addition, Ct‐values above the cut‐off, while not reproducible, were still within range of the limit of detection for the analysis. We were not able to confirm the presence of PMCV in weakly positive samples, probably due to limitations in sensitivity of the standard RT‐PCR analyses that were employed. Although the overall real‐time RT‐PCR and PCR results put together cannot be considered conclusive, they do suggest that care should be taken when assessing negative test results from sexual products of broodfish.

In six of the 14 egg batches tested, PMCV could be detected by PCR after disinfection, albeit at a lower prevalence than before disinfection. The biophysical properties of PMCV are not known, and the authors are not aware of any studies on the effect of disinfection on the virus thus far, mainly because there is no suitable protocol for cultivating PMCV for example in cell culture (Garseth et al., 2018). Thus, no attempts were made to ascertain whether the viral material detected by PCR in the present study was infective or not. The disinfection routine that was used is the one most commonly used in Norwegian salmon farming. The reduction in prevalence could be merely due to the mechanical rinsing off of the biological material present on the outside of the eggs. Based on the findings, we suggest that more thorough studies should be carried out to ascertain what disinfection measures should be used to reduce the risk of vertical transmission of PMCV.

Piscine myocarditis virus was detected in all batches at the eyed egg and shocked egg stages, of both cohorts. After hatching, PMCV was detected in a majority of the batches up until and including the 40 g stage. At the final sampling point, in presmolt, PMCV could no longer be detected in any of the batches we were following. However, PMCV was detected in fish sampled for tissue tropism from another batch in cohort X. With the sample size of 60 that we used in this sampling point, the detection limit is 4.9% prevalence (95% confidence), thus indicating that the PMCV could have been present even in the study batches, but at a lower level than we were able to detect.

The reduction in prevalence of PMCV with time in cohort X might suggest that the virus is not actively replicating within the progeny, and thus that they are either not actually infected but the PMCV detected is only from the surface of the fish, or that PMCV does not replicate in any considerable amount in these juvenile life stages of Atlantic salmon. In cohort Y, the prevalence of PMCV was relatively high at the final sampling point in two of the groups sampled.

The provided data of detection of PMCV in eggs and fry do not give any evidence for a true infection with replicating virus. In this study, we did not sequence the virus or measure early immune response markers for viral infection and at the 60 g sample stage we could not detect virus in the fish. In the PMCV disease challenge study by Timmerhaus et al. (Timmerhaus et al., 2011), the authors detected activation in six gene sets associated with immune responses, 2 weeks after infection of 50 g fish. However, in this study the fish were infected with a high dose of virus in post‐smolts and it may be that lower infection doses do not trigger an immune response before the virus level reach a trigger level. Although we did not find virus after 60 g in our study, we know from field reports that PMCV is detected in a limited number of smolt groups prior to sea‐transfer in hatcheries using only fresh water. This suggests that the virus can be retained in the population throughout the entire hatchery phase. Nevertheless, there have been no reports of any CMS outbreaks in hatcheries, neither any CMS development in challenge tests conducted on fry nor presmolts. This may imply either that (a) the virus replicates only at a very low level in juvenile fish; (b) the fish lack the ability to promote an immune response against PMCV in juvenile stages; or (c) the virus detected is incomplete, and the PCR results only detect fragments of the virus.

Infection of live virus at high levels is known to trigger an immune response, which may give protection later in life. To our knowledge, there has been no field or experimental studies that have investigated the relation between presence of PMCV in juveniles and protection against CMS at later stages. Unpublished studies on field data on heart and skeletal muscle inflammation (HSMI) strongly suggest that even high presence of the associated piscine reovirus (PRV) in juveniles does not give any protection against HSMI after sea‐transfer (H. Takle, *personal communication*). Therefore, we suggest that presence of PMCV at low titre likely will not give any natural immune protection against CMS. Smolts transferred to sea with PMCV virus would rather have an increased likelihood of developing CMS later in life. Nevertheless, this topic needs to be studied explicitly to make any conclusions as it is known that CMS spreads horizontally (Bang Jensen et al., 2013; Fritsvold et al., 2009; Haugland et al., 2011).

In order to assess whether the transmission really did occur from the individual broodstock to their respective progeny through vertical transmission, sequences of PMCV from the broodstock and the fry could have been compared. However, in the present study, the samples were generally too small, so that there was not enough material left to perform sequencing after the RT‐PCR was done. In addition, the studies performed so far on phylogeny of PMCV have shown little potential for molecular tracing (Wiik‐Nielsen, Alarcón, Fineid, Rode & Haugland, 2013; Xu, Mikalsen, Munang’andu & Evensen, 2018). In planning of the study, we aimed to include a negative control, in order to make sure that any findings of PMCV in progeny were not simply due to cross‐contamination from the broodstock or from viral residues in the environment or equipment. Unfortunately, no such negative control was available from the population of broodstock tested. A possible solution would be to repeat the study in a laboratory facility where it is possible to control for these things. As the egg batches were disinfected immediately after fertilization and then kept in single incubators, we believe we have minimized the risk for such cross‐contaminations.

For this transmission route to be of any consequence in the production, it is imperative to determine how a potential vertical transmission of PMCV occurs. One way to investigate this could be to perform infection trials with homogenate from organs that have been aseptically extracted from the fish. Using homogenated, disinfected eggs could also help determine whether PMCV is transmitted on the outside or the inside of the eggs, and whether proper disinfection could reduce the risk of transmission. The one aquatic viral disease that has been studied most extensively when it comes to its potential for vertical transmission is *infectious pancreatic necrosis* (IPN). Since 1963, a large number of diverse studies have tried to ascertain how this virus is transmitted vertically, but there is still no consensus as to whether it occurs as an embryonal infection, through contaminated water entering the egg, or plainly by resistance to disinfection with iodophores (Munro & Midtlyng, 2011). For PMCV, any of these means of vertical transmission could be valid.

We tested eggs and progeny from different combinations of PMCV positive and negative males and females, in order to investigate whether any transmission occurred from the males or the females. In our study, we could not see any substantial difference between the batches originating from the different combinations. However, those groups from both cohorts, where no PMCV was found in milt, were the ones with the lowest prevalences after hatching. Whether this is just a coincidence, or actually an indication that transmission from the males is more important than from the females should be investigated. A lot less males than females are needed for production, so that it could be feasible to only use PMCV‐negative males to reduce the risk of transmission.

In our study, we applied a “worst‐case” approach, selecting the batches with highest prevalence in the previous testing for each following sampling point, since we wanted to assess whether it was at all possible to continue detecting PMCV. Therefore, the prevalence of PMCV in the regular hatcheries is probably lower than what we have demonstrated here.

The present study provides a valuable input into the ongoing investigation of whether PMCV can be transmitted vertically, in addition to the well‐known horizontal transmission pathway. Further studies are needed in order to ascertain whether the PCR‐products detected in progeny is viable and infective PMCV or just non‐infective viral fragments. This could for example be done in a study using PCR‐positive material from eggs or fry in a challenge trial.

A recent study performed within the same project has shown that PMCV might be much more widespread in the Norwegian population of farmed salmon than previously thought (Svendsen et al., 2019). In this study, PMCV was found in farms with no or little prior history of CMS. Some of these farms were situated in an area where the overall prevalence of CMS until very recently has been low (Hjeltnes et al., 2018). The virus was detected soon after transfer to the sea, thus indicating that either the infection pressure of PMCV in the marine environment is high, or that the smolt were already infected at the time of sea‐transfer. Thus, we suggest that further studies should try and ascertain the prevalence of PMCV in hatcheries and especially in presmolt.

In the meantime, farmers have several options for minimizing the risk of transfer of PMCV from broodstock to progeny, including screening of broodstock and aiming to use only those that are negative for PMCV or have low levels of virus. Additionally, better routines for disinfection of milt and roe should be explored, and smolt could be screened for PMCV before transfer to the sea.

## CONFLICT OF INTEREST

Author Stian Nylund is affiliated with Pharmaq Analytiq which offers screening for salmon viruses included PMCV.
